# Author Correction: Coordinated peptidoglycan synthases and hydrolases stabilize the bacterial cell wall

**DOI:** 10.1038/s41467-024-52570-5

**Published:** 2024-09-19

**Authors:** Huan Zhang, Srutha Venkatesan, Emily Ng, Beiyan Nan

**Affiliations:** Department of Biology, Texas A&M University, College Station, TX 77843 USA

Correction to: *Nature Communications* 10.1038/s41467-023-41082-3, published online 2 September 2023

The original version of the Supplementary Information associated with this Article contained an error in Fig. S3c, in which the images of *∆dacB* cells from Fig. 3c were inadvertently duplicated in Fig. S3c. The HTML has been updated to include a corrected version of the Supplementary Information.

## Supplementary information

Updated Supplementary information

