# Supplementary material for: Coordinated peptidoglycan synthases and hydrolases stabilize the bacterial cell wall
Source: Nat Commun. 2023 Sep 2;14:5357. doi: 10.1038/s41467-023-41082-3 (PMC10475089; doi:10.1038/s41467-023-41082-3)
Supplement: Supplementary file 1 — Supplementary Information [file 41467_2023_41082_MOESM1_ESM.pdf]

Supplementary Information

Coordinated peptidoglycan synthases and hydrolases stabilize the bacterial cell wall

Huan Zhang[1], Srutha Venkatesan[1], Emily Ng[1] and Beiyan Nan[1]*

[1] Department of Biology, Texas A&M University, College Station, TX 77843, USA.

These authors contributed equally: Huan Zhang, Srutha Venkatesan

* email: bnan@tamu.edu

Table S1. *M. xanthus* strains (all genetic modifications were made on chromosome).

M. xanthus strains	Source	Identifier
DZ2 (wild-type *M. xanthus* strain)	[2]	DZ2
ΔdacB	[3]	TM1142
dacB^{S75A}	This study	BN303
Δpbp1a1	This study	BN304
Δpbp1a2	This study	BN305
pbp1a2^{E97A}	This study	BN306
Δpbp1c	This study	BN307
Δpbp1a1 Δpbp1a2	This study	BN308
Δpbp1a1 pbp1c::kan	This study	BN309
Δpbp1a2 Δpbp1c	This study	BN310
Δ3 (Δpbp1a1 Δpbp1a2 pbp1c::kan)	This study	BN311
dacB-PAmCherry	This study	BN312
dacB^{S75A}*-PAmCherry::kan*	This study	BN313
pbp1a2^{E97A} *dacB-PAmCherry::kan*	This study	BN314
P_{van} *dacB-PAmCherry* (for DacB overexpression)	This study	BN315
pMR3629 (P_{van} vector control)	This study	BN316
Δpbp1a1 dacB-PAmCherry::kan	This study	BN317
Δpbp1a2 dacB-PAmCherry::kan	This study	BN318
Δpbp1c dacB-PAmCherry::kan	This study	BN319
pbp1a1-PAmCherry	This study	BN320
pbp1a2-PAmCherry	This study	BN321
pbp1a2^{E97A}*-PAmCherry::kan*	This study	BN322
pbp1c-PAmCherry	This study	BN323
ΔdacB pbp1a1-PAmCherry::kan	This study	BN324
ΔdacB pbp1a2-PAmCherry::kan	This study	BN325
ΔdacB pbp1c-PAmCherry::kan	This study	BN326

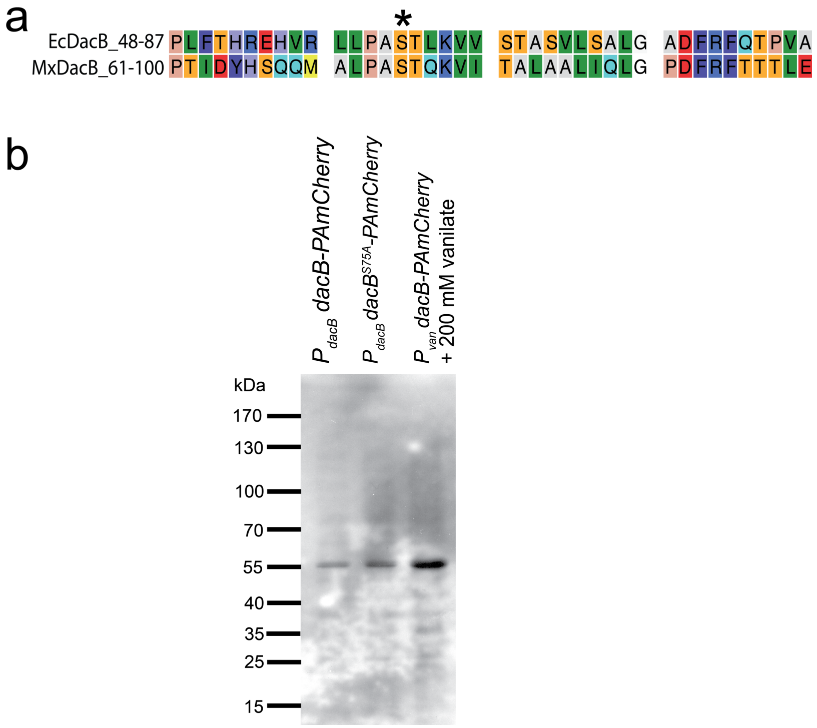

Fig. S1. DacB-PAmCherry and DacBS75A-PAmCherry accumulate as full-length proteins. a) Sequence alignment between the DacB homologs in *M. xanths* (MxDacB) and *E. coli* (EcDacB). The catalytic active serine residue is marked by *. **b)** Immunoblotting using *M. xanthus* cell lysates and an anti-mCherry antibody.

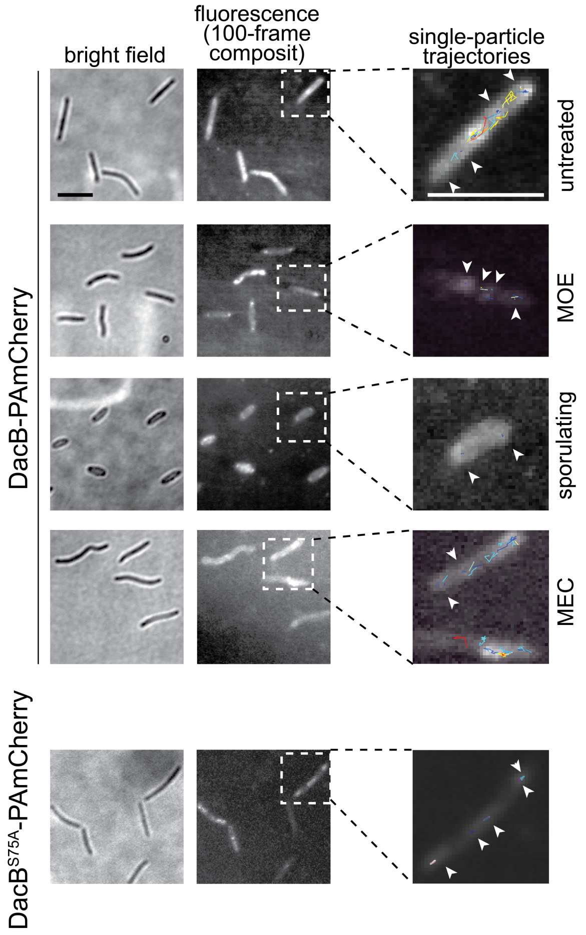

Fig. S2. Overall distribution and single-particle trajectories of DacB-PAmCherry and DacB^{S75A}-PAmCherry. Overall distribution of single molecules is displayed using the composite of 100 frames. Single-particle trajectories were generated from 100 frames using the TrackMate[1] plugin in ImageJ. Individual trajectories are distinguished by colors. White arrows point to stationary particles. MOE, moenomycin, 4 μg/ml; MEC, mecillinam, 100 μg/ml. Sporulation was induced by 1M glycerol and cells were imaged after 1 h of induction. Scale bars, 5 μm.

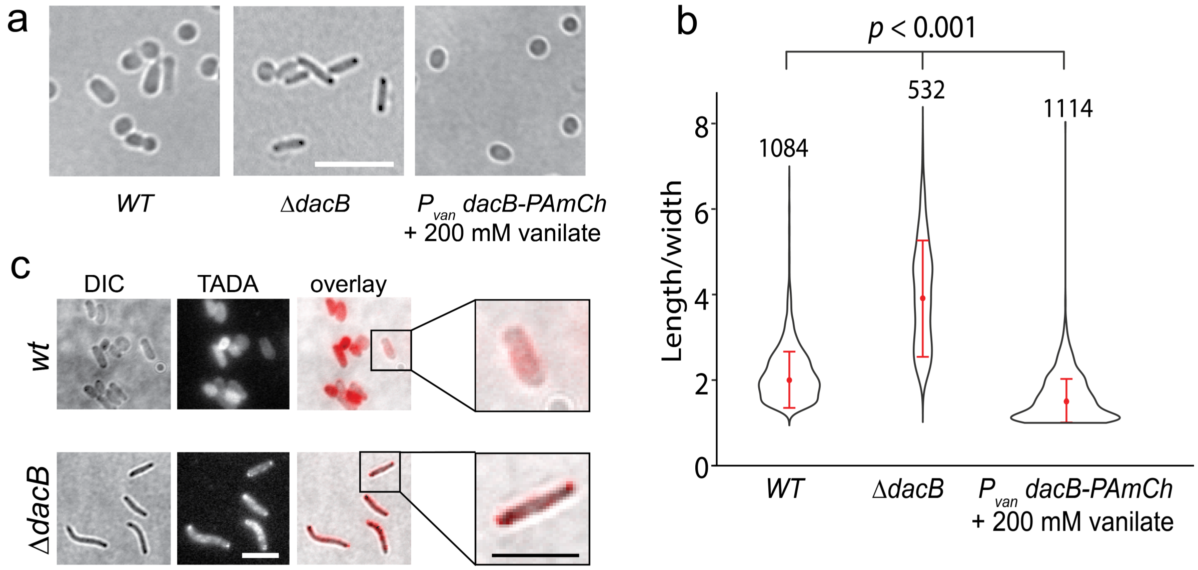

Fig. S3. DacB is a major PG hydrolase that dismantles rod shape during the sporulation of *M. xanthus*. a) The absence and overexpression of DacB delays and accelerates the sporulation of *M. xanthus*, respectively. Phase contrast images of cells after 2-h of glycerol induction are shown. **b)** The progress of sporulation is quantified by the length/width ratio of cells after 2-h of glycerol induction. Whiskers indicate the 25th - 75th percentiles and red dots the median. The total number of cells analyzed is shown on top of each bar. *p* values were calculated using a one-way ANOVA test between unweighted, independent samples. **c)** While wild-type cells lose TADA signal near evenly during sporulation, the Δ*dacB* cells retain TADA at their poles. Scale bars, 5 μm.

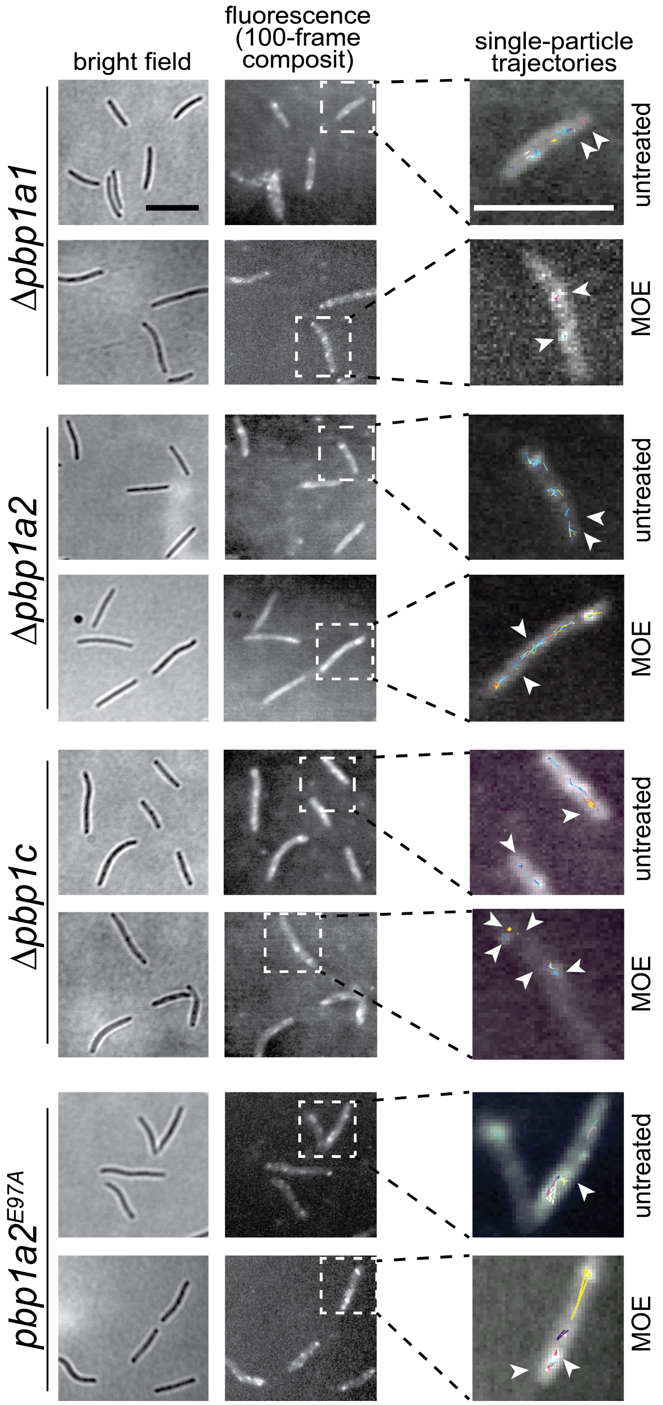

Fig. S4. Overall distribution and single-particle trajectories of DacB-PAmCherry in the backgrounds of aPBP mutants. Overall distribution of single molecules is displayed using the composite of 100 frames. Single-particle trajectories were generated from 100 frames using the TrackMate[1] plugin in ImageJ. Individual trajectories are distinguished by colors. White arrows point to stationary particles. MOE, moenomycin, 4 μg/ml. Scale bars, 5 μm.

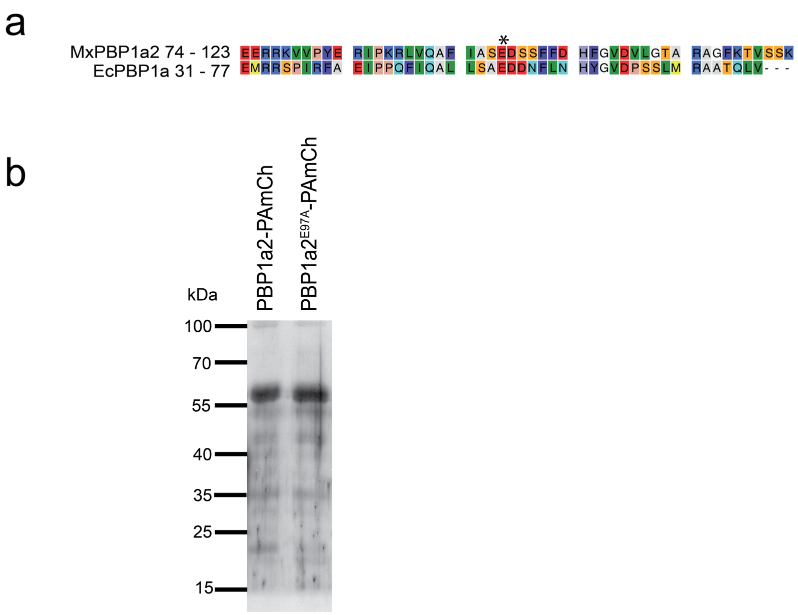

Fig. S5. The expression of PBP1a2^{E97A} in *M. xanthus*. **a)** Sequence alignment between *M. xanths* PBP1a2 (PBP1a2) and *E. coli* PBP1a (EcPBP1a). The catalytic active glutamate residue is marked by *. **b)** PBP1a2^{E97A} expresses as a stable protein in *M. xanthus*. Its stability was tested using cell lysate of a strain that expresses PBP1a2^{E97A}-PAmCherry as the sole source of PBP1a2 and an anti-mCherry antibody.

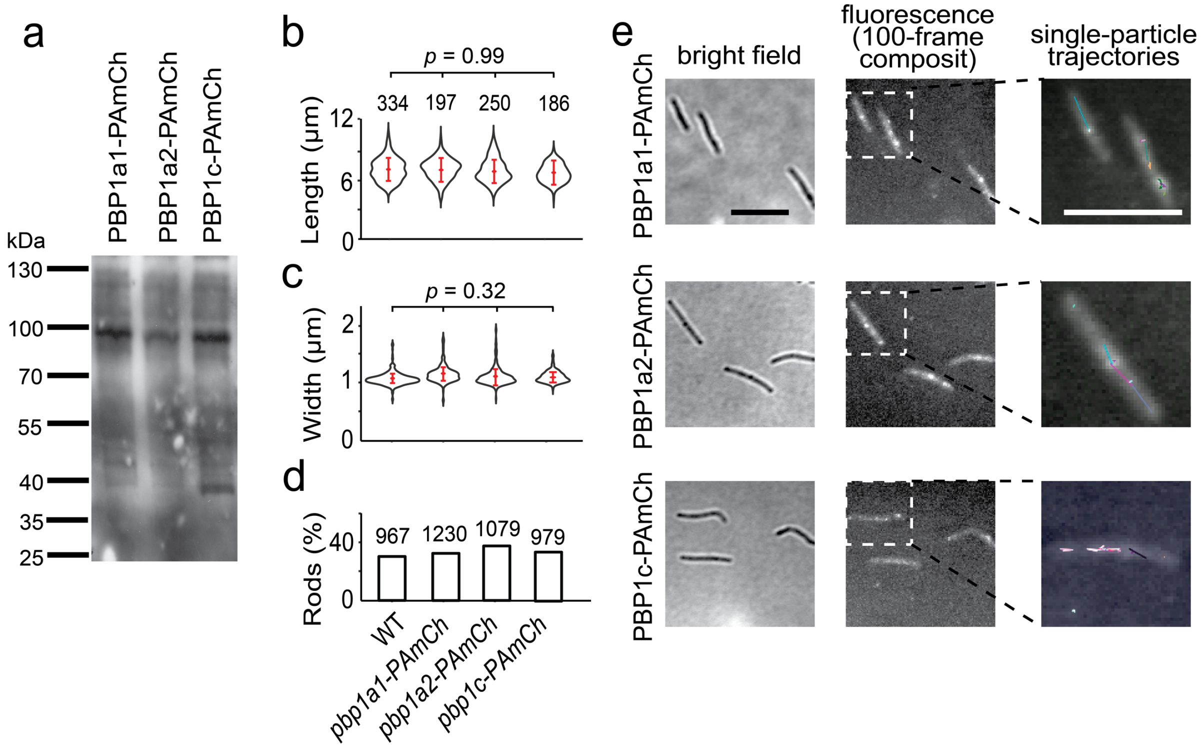

Fig. S6. Overall distribution and single-particle trajectories of PAmCherry-labeled aPBPs. a) All three PAmCherry-labeled aPBPs accumulate as full-length proteins. Their stability was tested using cell lysate of the strains that each express one PAmCherry-labeled aPBP and an anti-mCherry antibody. A low molecular weight band is visible in the lane of PBP1c-PAmCherry. However, its molecular weight, ~40 kDa, does not match degraded PAmCherry (~25 kDa). **b-d)** PAmCherry label on aPBPs does not affect the length (**b**), width (**c**), and resistance against moenomycin (8 µg/ml, 2 h) (**d**). Whiskers indicate the 25th - 75th percentiles and red dots the median. The total number of cells analyzed is shown on top of each bar. *p* values were calculated using the Student paired t test with a two-tailed distribution. **e)** Overall distribution of single molecules is displayed using the composite of 100 frames. Single-particle trajectories were generated from 100 frames using the TrackMate[1] plugin in ImageJ. Individual trajectories are distinguished by colors. Scale bars, 5 µm.

Supplementary References

1 Ershov, D. *et al.* TrackMate 7: integrating state-of-the-art segmentation algorithms into tracking pipelines. *Nat Methods* **19**, 829-832 (2022). https://doi.org:10.1038/s41592-022-01507-1

2 Campos, J. M., Geisselsoder, J. & Zusman, D. R. Isolation of bacteriophage MX4, a generalized transducing phage for *Myxococcus xanthus*. *J Mol Biol* **119**, 167-178 (1978).

3 Zhang, H. *et al.* Establishing rod shape from spherical, peptidoglycan-deficient bacterial spores. *Proc Natl Acad Sci U S A* **117**, 14444-14452 (2020). https://doi.org:10.1073/pnas.2001384117
