# Supplementary material for: Coordinated peptidoglycan synthases and hydrolases stabilize the bacterial cell wall
Source: Nat Commun. 2023 Sep 2;14:5357. doi: 10.1038/s41467-023-41082-3 (PMC10475089; doi:10.1038/s41467-023-41082-3)
Supplement: Supplementary file 3 — Reporting Summary [file 41467_2023_41082_MOESM3_ESM.pdf]

nature portfolio

Corresponding author(s): Beiyan Nan

Last updated by author(s): Jul 11, 2023

Reporting Summary

Nature Portfolio wishes to improve the reproducibility of the work that we publish. This form provides structure for consistency and transparency in reporting. For further information on Nature Portfolio policies, see our Editorial Policies and the Editorial Policy Checklist.

Statistics

For all statistical analyses, confirm that the following items are present in the figure legend, table legend, main text, or Methods section.

n/a	Confirmed	
☐	☒	The exact sample size (*n*) for each experimental group/condition, given as a discrete number and unit of measurement
☐	☒	A statement on whether measurements were taken from distinct samples or whether the same sample was measured repeatedly
☐	☒	The statistical test(s) used AND whether they are one- or two-sided<br>*Only common tests should be described solely by name; describe more complex techniques in the Methods section.*
☒	☐	A description of all covariates tested
☒	☐	A description of any assumptions or corrections, such as tests of normality and adjustment for multiple comparisons
☒	☐	A full description of the statistical parameters including central tendency (e.g. means) or other basic estimates (e.g. regression coefficient) AND variation (e.g. standard deviation) or associated estimates of uncertainty (e.g. confidence intervals)
☐	☒	For null hypothesis testing, the test statistic (e.g. *F*, *t*, *r*) with confidence intervals, effect sizes, degrees of freedom and *P* value noted<br>*Give P values as exact values whenever suitable.*
☒	☐	For Bayesian analysis, information on the choice of priors and Markov chain Monte Carlo settings
☒	☐	For hierarchical and complex designs, identification of the appropriate level for tests and full reporting of outcomes
☒	☐	Estimates of effect sizes (e.g. Cohen's *d*, Pearson's *r*), indicating how they were calculated

Our web collection on statistics for biologists contains articles on many of the points above.

Software and code

Policy information about availability of computer code

Data collection	Nikon NIS-Elements AR 5.30.05 64-bit
Data analysis	The codes used for cell dimension measurement and single-particle dynamic analysis are described in ref. 23 and deposited in the GitHub repository (ref. 45, https://doi.org:10.5281/zenodo.8234126).

For manuscripts utilizing custom algorithms or software that are central to the research but not yet described in published literature, software must be made available to editors and reviewers. We strongly encourage code deposition in a community repository (e.g. GitHub). See the Nature Portfolio guidelines for submitting code & software for further information.

Data

Policy information about availability of data

All manuscripts must include a data availability statement. This statement should provide the following information, where applicable:

- Accession codes, unique identifiers, or web links for publicly available datasets
- A description of any restrictions on data availability
- For clinical datasets or third party data, please ensure that the statement adheres to our policy

All data supporting the findings of this study are available within the paper and its supplementary information. Due to their large sizes, raw microscopy images and videos are available from the corresponding author upon request. Processed cell dimension and single-particle tracking data are provided in the Source Data file, which is provided with this paper.

Research involving human participants, their data, or biological material

Policy information about studies with human participants or human data. See also policy information about sex, gender (identity/presentation), and sexual orientation and race, ethnicity and racism.

Reporting on sex and gender	N/A
Reporting on race, ethnicity, or other socially relevant groupings	N/A
Population characteristics	N/A
Recruitment	N/A
Ethics oversight	N/A

Note that full information on the approval of the study protocol must also be provided in the manuscript.

Field-specific reporting

Please select the one below that is the best fit for your research. If you are not sure, read the appropriate sections before making your selection.

- [x] Life sciences
- [ ] Behavioural & social sciences
- [ ] Ecological, evolutionary & environmental sciences

For a reference copy of the document with all sections, see nature.com/documents/nr-reporting-summary-flat.pdf

Life sciences study design

All studies must disclose on these points even when the disclosure is negative.

Sample size	OD values were determined from six replicates. No statistical methods were used to predetermine sample sizes for cell morphology measurements and single-particle tracking. Sample sizes were determined based on our expertise in bacterial cell biology and single-particle microscopy. For cell morphology measurements and single-particle tracking, data were collected from large numbers of cells (minimum 150 cells for morphology measurements and 100 cells for single-particle tracking). For each experiment, the results were confirmed by two additional biological replicates.
Data exclusions	No data were excluded.
Replication	All experiments were replicated by at least two biological replicates. All replications produced similar results. The nature and number of replication is indicated in figure legends. Microscopy images presented are representative of the whole population in each condition.
Randomization	As there is no allocation to subgroups, randomization is not applicable.
Blinding	Two researchers ran analysis algorithms independently, one of them doing so in a blind fashion.

Reporting for specific materials, systems and methods

We require information from authors about some types of materials, experimental systems and methods used in many studies. Here, indicate whether each material, system or method listed is relevant to your study. If you are not sure if a list item applies to your research, read the appropriate section before selecting a response.

Materials & experimental systems

n/a	Involved in the study	
[ ]	[x]	Antibodies
[x]	[ ]	Eukaryotic cell lines
[x]	[ ]	Palaeontology and archaeology
[x]	[ ]	Animals and other organisms
[x]	[ ]	Clinical data
[x]	[ ]	Dual use research of concern
[x]	[ ]	Plants

Methods

n/a	Involved in the study	
[x]	[ ]	ChIP-seq
[x]	[ ]	Flow cytometry
[x]	[ ]	MRI-based neuroimaging

Antibodies

Antibodies used	The expression and stability of PAmCherry-labeled proteins were determined by immunoblotting using an anti-mCherry antibody (Rockland Immunochemicals, Inc., Catlog # 600-401-P16, Lot 46705, 1:2,000 dilution) and a Goat anti-Rabbit IgG (H+L) Secondary

	Antibody, HRP (ThermoFisher Scientific, catalog # 31460, 1:10,000 dilution).
Validation	the specificity of the antibody was validated by the manufacture (https://www.rockland.com/categories/primary-antibodies/anti-mcherry-antibody-pre-adsorbed-600-401-P16/) and confirmed by our experiments.
